# Comparing emergency department visits 10-year apart at a tertiary care center in Lebanon

**DOI:** 10.1097/MD.0000000000035194

**Published:** 2023-09-29

**Authors:** Tharwat El Zahran, Lara Ghandour, Anwar Chami, Najat Saliba, Eveline Hitti

**Affiliations:** a Department of Emergency Medicine, Faculty of Medicine, American University of Beirut Medical Center, Beirut, Lebanon; b ALESOPI Consulting, Paris, France; c Department of Chemistry, Faculty of Arts and Sciences, American University of Beirut, Beirut, Lebanon.

**Keywords:** cardiovascular diseases, communicable diseases, double burden of diseases, emergency department, low-middle income countries, non-communicable diseases

## Abstract

Presentations to the emergency department (ED) are growing worldwide. With the increasing risk factors of non-communicable disease (NCD) and communicable diseases (CD) in low- and middle-income countries, it is crucial to understand how ED presentations are changing with time to meet patients’ needs and allocate acute care resources. The aim of this study is to compare the changes in patient and diseases characteristics over 2 time periods 10 year apart at the largest tertiary care center in Lebanon. This was a retrospective descriptive study of patients presenting to the ED at a large tertiary care center in 2009/2010 and 2018/2019. The discharge diagnoses were coded into Clinical Classification Software codes. We used descriptive statistics, odds ratios (OR), and non-parametric test to compare the different diagnoses. The total number of ED visits increased by 33% from 2009/2010 to 2018/2019. The highest increase rate was among patients older than 65 years (2.6%), whereas the percentage of pediatric patients decreased from 30.8% to 25.3%. ED presentations shifted from NCD to CD. A shift in the discharge diagnoses was also noted within age groups, specifically a shift in cardiovascular diseases to a younger age. Our study suggests that the role of the ED is changing and moving towards treating the aging population and CD. There is a need to invest and mitigate CD, better allocate resources to accommodate the aging population, focus on awareness campaigns targeting early detection of cardiovascular diseases and modifying its risk factors.

## 1. Introduction

Presentations to the emergency department (ED) are growing worldwide.^[1]^ During the past 2 decades, the annual rate of ED visits increased by 50% and exceeded population growth in the USA.^[2]^ The trend in ED visits has been inconsistent across diseases and patient characteristics.^[3,4]^ Patients are presenting to the ED for a wide range of diseases such as acute injury or illness, life-threatening emergencies, as well as non-urgent conditions. Several factors affect ED visits over time such as changes in socioeconomic factors, insurance coverage, healthcare system, and access to care.^[5,6]^

With the growing complexity of health systems and increasing need for ED services, it is crucial to trace and comprehend annual trends in the ED to strengthen emergency care, its preparedness, and management.^[7–9]^ However, this concern is ignored in several low- and middle-income countries (LMIC), even though the burden of emergency diseases is high in those countries.^[8,10]^ In fact, in LMIC basic information and national surveillance data on emergency care are limited due to incomplete medical records, absence of national data, and inability to conduct studies in some institutions. The absence of those factors negatively affects policymaking, patient care, training programs, and resources allocation.^[10,11]^

Previous studies in LMIC showed a double burden of diseases in the ED setting, including both non-communicable disease (NCD) and communicable diseases (CD). This was further studied in the ED setting at the largest hospital in Lebanon and the results confirmed that patients suffered from the double burden of NCDs and CDs.^[11]^ In Lebanon, the risk factors for NCDs have been rising during the last decade such as physical inactivity, diabetes, obesity and smoking.^[12]^ On the other hand, Lebanon has been experiencing a surge in refugees, power outages and air pollution, contributing to the increase in risk factors for CD.^[11,13–15]^ Therefore, it is crucial to understand and compare how ED visits and characteristics are changing with time as this will help design policies and training programs to meet patients’ needs and direct the allocation of acute care resources. Our study aims to compare how patient and disease characteristics have changed over 10 years at the largest tertiary care center in Lebanon.

## 2. Material and methods

### 2.1. Study design and setting

This study was a retrospective descriptive study that aimed to assess and compare patient and disease characteristics of ED visits at a tertiary care center in Lebanon during 2009/2010 and 2018/2019. The study was approved by the Institutional Review Board at the American University of Beirut (reference number: BIO-2019-049). The study was performed at the largest tertiary care center in Beirut, Lebanon. AUBMC is a 358-bed tertiary care center which receives 25,000 inpatient hospital admissions annually, and approximately 55,000 ED visits. The ED is divided into 3 sections (High Acuity, Low Acuity, and Pediatrics). The ED medical staff includes American Board-certified/eligible Emergency Medicine (EM) physicians and non-EM physicians who have extensive experience in emergency care as well as a mix of EM and non-EM resident rotators. While AUBMC has a transfer center it does not oversee the transport services which depend on a mix of governmental and non-governmental pre-hospital transport teams.

Due to the retrospective chart review nature of the study and the large number of patients to be enrolled, the Institutional Review Board at AUB approved to waive the informed consent for participation since it will make this study impracticable.

This study conforms with the Strengthening the Reporting of Observational Studies in Epidemiology guidelines and a complete checklist has been uploaded as Supplemental table. http://links.lww.com/MD/K36

### 2.2. Selection of participants

The study population included all patients currently living in Lebanon and presenting to the ED at AUMBC from July 01, 2009 to July 31, 2010 as well as November 01, 2018 to November 30, 2019, hereafter referred to as the year 2009/2010 and 2018/2019. The data included both adult and pediatric patients with all acuity levels. Due to resource limitations in the medical record department in our facility, the most recent fully coded year for ED visits was July 2009 to July 2010. Moreover, EPIC (Electronic Health Records) was implemented in our facility in November 2018. To avoid any missing and uncoded data, timeframes which have complete data records were selected: July 2009 to July 2010 and November 2018 to November 2019.

### 2.3. Study measures

The primary data collection was extracted from the electronic medical records of all patients presenting to the ED at AUMBC during 2009/2010 and 2018/2019. The variables collected include basic socio-demographic information such as age (0–5, 6–18, 19–44, 45–64, 65+), gender (male/female), and clinical characteristics (disposition and discharge diagnosis).

As for the discharge diagnoses, they were coded by the providers, extracted and validated by the trained medical records staff according to the 9th and 10th revision of the International Statistical Classification of Diseases and Related Health Problems (ICD-9/10-CM). Professional fee reimbursement in Lebanon is not based on diagnostic coding system but rather on a flat fee per visit. The medical records department completes the visit coding to generate data used for administrative decisions/records keeping. The coding personnel have all received formal ICD-9/10 coding training. Furthermore, even post-electronic health record (EHR) implementation, while the EHR includes a module that facilitates coding for physicians on entry of the discharge diagnosis, the medical records team continues to complete quality checks on this process.

There are approximately 69,000 ICD-10-CM codes and 14,000 ICD-9-CM codes for diagnosis. For unified coding and more manageable analysis, we used the Clinical Classification Software (CCS) codes.^[16]^ The CCS codes are 285 single level codes that collapse ICD 9/10 CM codes into a smaller number of clinically meaningful categories that can be more useful for presenting descriptive statistics than individual ICD 9/10 codes. The CCS for ICD 9/10 is one of the databases and software tools developed by the Healthcare Cost and Utilization Project, a Federal-State-Industry partnership sponsored by the Agency for Healthcare Research and Quality. Healthcare Cost and Utilization Project databases, tools, and software inform decision making at the national and community levels. For example, the CCS code for esophagus cancer is CCS12, it includes the following ICD9 Codes (1500-1509, 2301, V1003) and ICD10 codes (C153-C159, C49A1, D001, Z8501).^[16]^

### 2.4. Statistical analysis

This study aimed to assess and compare the ED visit characteristics during 2009/2010 and 2018/2019. The analysis was conducted in R and figures were produced using the package ggplot2.^[17]^ Descriptive statistics were summarized by compiling counts and related fractions over age, gender, and dispositions. OR and a 95% confidence interval (CI), were computed using Schratz, 2017 to evaluate the change in frequency of each diagnosis group with the 2 timepoints as a measure of association.^[18]^ A significance level of *P* < 0.05 was considered indicative of statistical significance. A nonparametric test of the equality of continuous, one-dimensional probability distributions (the Kolmogorov–Smirnov test) was used to compare the age distribution of different diagnosis groups admissions over the 2 years. Graphical methods such as hybrid bar charts and Cleveland dot plots were used to visualize the evolution of OR for different diagnosis groups. Additionally, Kernel Density Plots were used to visualize the distribution of admissions over patient age (using a difference of “area under the curve” calculation).

## 3. Results

### 3.1. Patient demographics

During the study period, the number of ED presentations rose among all patients’ subgroups. The total number of ED visits increased from 42,109 to 56,015 visits (33% increase) between 2009/2010 and 2018/2019. Percentages of ED visits to AUBMC from the total population would therefore be 0.85% and 0.94%, respectively, with a difference in proportion between the 2 population of −0.091% (95% CI [−0.10, −0.07], *P* < .001).

The majority of ED visits were for patients aged 19 to 44 years (38.1% and 40.0% in 2009/2010 and 2018/2019, respectively) and the highest increase rate was among patients older than 65 years (increased from 14.2% to 16.8%). The percentage of pediatric patients presenting to the ED decreased from 30.8% to 25.3% over 10 years. The rate remained approximately stable for gender, where male patients were more likely to present to the ED in both years, 52% and 50.7% in 2009/2010 and 2018/2019, respectively.

From 2009/2010 to 2018/2019, there was a significant change in the patients’ disposition. The number of patients admitted to the hospital following the ED visit increased from 14.3% to 20.2% in 2009/2010 and 2018/2019, respectively. The proportion of patients treated and discharged home decreased from 85.5% to 79.6%. There was no change in the rate of death (Table 1).

**Table 1:** Emergency department patients and visits characteristics, in 2009/2010 and 2018/2019.

Characteristics	All ED visits N (%)		
	2009/2010	2018/2019	*P* value
Total ED visits	42,109 (100)	56,015 (100)	
Age			
0–5	6053 (14.4)	7012 (12.5)	.07
6–18	6887 (16.4)	7148 (12.8)	
19–44	16,030 (38.1)	22,431 (40.0)	
45–64	7161 (17.0)	10,009 (17.9)	
>65	5978 (14.2)	9413 (16.8)	
Sex			
Male	21,885 (52.0)	28,403 (50.7)	.62
Female	20,222 (48.0)	27,609 (49.3)	
Disposition			
In-patient admission	6010 (14.3)	11,306 (20.2)	**<.001**
Discharged	35,996 (85.5)	44,611 (79.6)	
Death	103 (0.2)	98 (0.2)	

A *P*-value < 0.05 is considered significant.

ED = emergency department.

### 3.2. Diagnosis prevalence

**Figure 1.**
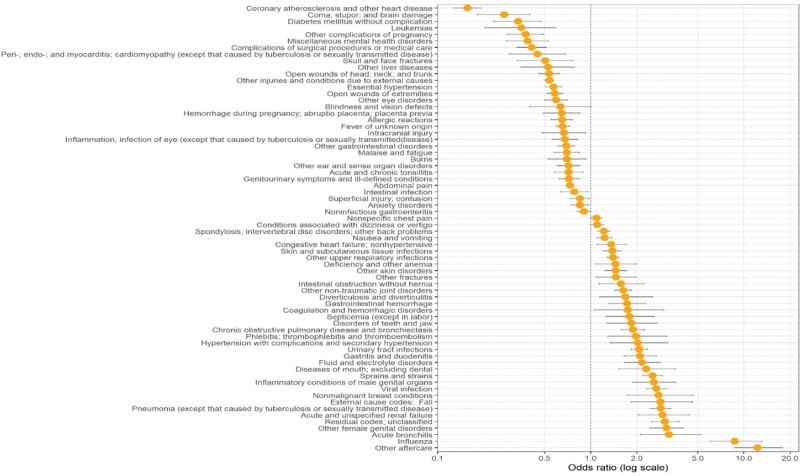
Comparison between 2009/2010 and 2018/2019 discharge diagnoses.

The comparison between the 2 timepoints is described as the OR of each diagnosis in reference to 2018/2019. An OR > 1 means that the diagnosis was more prevalent in 2018/2019 and an OR < 1 means the diagnosis was more prevalent in 2009/2010. Figure 1 shows the diagnoses with more than 20 admissions in each year and whose OR has a significance level of *P* value < .05.

Results show that, in comparison to 2009/2010, 2018/2019 patients were more likely to be discharged for influenza (OR = 8.88 [6.12–13.5]), acute bronchitis (OR = 3.20 [2.08–5.16]), pneumonia (OR = 2.82 [2.43–3.28]), viral infection (OR = 2.64 [2.28–3.07]), UTIs (OR = 2.05 [1.82–2.32]), as well as hypertension with complications, and secondary hypertension (OR = 2.00 [1.32–3.14])., ‘The 2018/2019 patients were less likely (and the 2009/2010 patients therefore more likely) to be discharged with coronary atherosclerosis and other heart diseases (OR = 0.15 [0.12–0.18]), diabetes mellitus without complication (OR = 0.32 [0.22–0.46]), leukemias (OR = 0.35 [0.20–0.58]), other complications of pregnancy (OR = 0.36 [0.28–0.47]) and miscellaneous mental health disorders (OR = 0.38 [0.27–0.51]).

### 3.3. Age at admission

**Figure 2.**
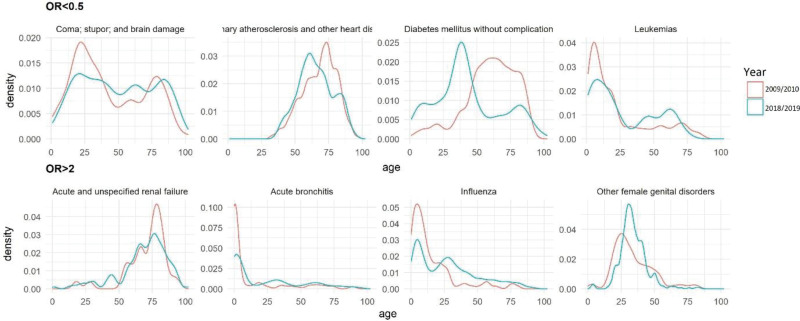
Age density plots of discharge diagnoses showing most change in distribution of age density in 2009/2010 and 2018/2019.

Figure 2 presents age density plots comparing 8 discharge diagnoses that showed the difference in their age distribution in each of the 2 years. These discharge diagnoses were identified by having a Kolmogorov–Smirnov *P* value < .05 and then ranked according to the most difference in the area under the curve over 2 sets of prevalence: more prevalent in 2009/2010 (OR of diagnosis <0.5) and more prevalent in 2018/1019 (OR > 2).

Patients were more likely to present to the ED with coma, stupor, or brain damage in 2009/2010 with a bimodal with 2 peaks, around 25 and 75 years of age. This curve was considerably flattened in 2018/2019 indicating a more even age distribution. Coronary atherosclerosis was another major diagnosis prevalent in 2009/2010. Its distribution, which was slightly flatter in 2018/2019, showed a shift and peak to the left indicating that recently patients are diagnosed with coronary atherosclerosis at a younger age (60 years vs 73 years in 2018/2019 and 2009/2010, respectively). Moreover, patients were more likely to be diagnosed with diabetes mellitus (without complication) in 2009/2010 compared to 2018/2019, however, in 2018/2019, the distribution peaked 25 years earlier. Around age 10, the major peak for leukemia was similar between the 2 timepoints however a smaller secondary peak around age 60 was noticed for 2018/2019.

As for the discharge diagnoses with more prevalence in 2018/2019 showing the most difference in age distribution across the 2 study years, acute and unspecific renal failure, acute bronchitis, influenza, and female genital disorders stand out. The age distribution in the first 3 diagnoses showed in 2018/2019 a more even distribution and lower peaks at the age of 75 for acute and unspecific renal failure, and in the pediatric population for acute bronchitis and influenza. Noted that influenza also showed bimodal peaks near age 7 and 25 in 2018/2019. On the other hand, the discharge diagnosis of female genital disorders had a pertinent peak in 2018/2019 compared to 2009/2010.

### 3.4. Change in top diagnoses

The top 5 diagnoses among the different age groups in 2009/2010 and 2018/2019 are ranked in Tables 2 and 3, respectively. The most common diagnoses among patients younger than 5 years in 2009/2010 were: fever, presentations related to other injuries and conditions due to external causes, and upper respiratory infections. In 2018/2019: viral infection, fever and, other upper respiratory infections topped the diagnosis.

**Table 2:** Number of patients and ranking of the top 5 most common ED discharge diagnoses by age groups in 2009/2010. Presented as N (Ranking).

	0–5 yr		6–18 yr		19–44 yr		45–64 yr		65 + yr	
	N	Rank	N	Rank	N	Rank	N	Rank	N	Rank
Fever of unknown origin	1271	1	891	2	608	5				
Other injuries and conditions due to external causes	372	2	930	1	1903	1	568	1	278	3
Other upper respiratory infections	366	3	313	4	719	3				
Other lower respiratory infections	327	4	292	5					228	5
Open wounds	325	5								
Abdominal pain			386	3	1446	2	541	2	272	4
noninfectious gastroenteritis					628	4				
nonspecific chest pain							449	3		
Spondylosis, intervertebral disc disorder, other back pain							252	4		
Essential hypertension							240	5	299	2
Coronary atherosclerosis and other heart diseases									321	1

**Table 3:** Number of patients and ranking of the top 5 most common ED discharge diagnoses by age groups in 2018/2019. Presented as N (Ranking).

	0–5 yr		6–18 yr		19–44 yr		45–64 yr		65 + yr	
	N	Rank	N	Rank	N	Rank	N	Rank	N	Rank
Viral infection	964	1	404	3						
Fever of unknown origin	729	2	321	5						
Other upper respiratory infections	726	3	481	2	1309	3				
Otitis media and related conditions	433	4								
Other injuries and conditions due to external causes	326	5	706	1	1374	2	487	3	380	3
Abdominal pain			351	4	1583	1	611	2		
noninfectious gastroenteritis					877	4				
Headache, including migraine					789	5				
nonspecific chest pain							651	1	352	4
Spondylosis, intervertebral disc disorder, other back pain							442	4		
Other lower respiratory infections							382	5	527	1
Pneumonia									462	2
Urinary tract infection									350	5

For children aged 6 to 18 years, the top-ranked ED diagnoses in 2009/2010 were other injuries and conditions due to external causes, fever, and abdominal pain. In 2018/2019, the ranking was similar for the first diagnosis but changed to other upper respiratory infections and viral infections for the second and third. For older patients (19–44 years), other injuries and conditions due to external causes also ranked first, followed by abdominal pain, upper respiratory infections, and noninfectious gastroenteritis (4) in 2009/2010. Meanwhile, in 2018/2019 the diagnoses were almost consistent, with abdominal pain being listed before other injuries and conditions due to external causes. For patients aged 45 to 64 years, chest pain ranked first in 2018/2019, compared to ranking third in 2009/2010. Other injuries and conditions due to external causes and abdominal pain were both in the top diagnoses in 2009/2010 and 2018/2019. Circulatory diseases (Hypertension and Coronary atherosclerosis and other heart diseases) were the top 2 diagnoses in patients older than 65 in 2009/2010, however, in 2018/2019, respiratory diseases (pneumonia and other lower respiratory diseases) ranked among the top 5 diagnoses.

## 4. Discussion

This is the first study to compare the changes in ED visits and patients’ characteristics 10 years apart at the largest tertiary care center in Lebanon, setting baseline surveillance data in a LMIC. The study found that when comparing 2009/2010 to 2018/2019, the number of ED visits increased especially among middle and older patients. ED presentations shifted from NCD to CD, particularly among older patients. This highlights the changing role of the ED in the healthcare system in LMIC, where EDs are moving towards treating the aging population and CD.

Results show that the overall presentations to the ED increased, especially among middle- aged and older patients. In particular, the highest increase was among patients older than 65 years (by 2.6%), whereas the percentage of pediatric patients presenting to the ED decreased from 30.8% to 25.3%. In addition, the admission rate increased from 14.3% to 20.2%, while the treat and release rate decreased from 85.5% to 79.6%. This is mainly attributed to the demographic changes in the Lebanese population. A decrease among the age group 0 to 14 years from 26.5% in 2009 to 25.6% in 2019 and an increase among citizens older than 65 years from 6.27% to 7.27% from 2009 to 2019 has been reported.^[19,20]^ The population-based data suggest that Lebanon is becoming an aging country which significantly affects the use of healthcare services, especially EDs,^[21]^ which is confirmed by the surge in annual health expenditure (as a percentage of GDP) from 6.99% in 2009 to 8.35% in 2018.^[22]^

Interestingly, a shift from NCD to CD was observed between the 2 timeframes. In 2009/2010 patients tended to present to the ED for diabetes mellitus, leukemia, and atherosclerosis, whereas in 2018/2019 infectious related diseases predominated such as pneumonia, bronchitis, and influenza. Even though reports from LMIC reported double burden of diseases in the ED,^[11]^ our study showed that ED diagnoses are shifting from NCD towards CD. In comparison to other Middle Eastern and North African countries many NCDs, including cardiovascular diseases, chronic respiratory diseases, digestive diseases, have been mitigated except for diabetes and kidney diseases. Yet, several infectious diseases worsened over the years such as HIV/AIDS and STIs, and enteric infections.^[23]^ The results are also in line with high-income countries (HIC) such as the US, where coronary atherosclerosis, and heart diseases have decreased recently, whereas diagnoses related to infectious and parasitic diseases rose.^[24,25]^ Moreover, global health data showed that there was an improvement in the majority of NCD globally. This suggests that many healthcare systems and governments are prioritizing and allocating resources to reduce NCD. In Lebanon, extensive effort and initiatives from the Lebanese public health ministry and the World Health Organization have been put to prevent, detect, and manage cardiovascular diseases and other NCD such as diabetes, cancer, and lung diseases^[26]^ which contributed to the mitigation and early detection of NCD.

Moreover, a shift in the discharge diagnoses was also noted within age groups. In both years, patients younger than 18 years mainly presented to the ED for fever, injuries, abdominal pain, and respiratory infections. These results are in line with other LMIC^[27,28]^ and HIC.^[29,30]^ As for patients aged 19 and 44 years, there was no change in the discharge diagnoses in both years, they presented for injuries, abdominal pain, respiratory infections, and noninfectious gastroenteritis. Interestingly, ED injury-related diagnoses decreased in this age group, which is similar to ED trends in HIC.^[24]^ This emphasizes that the ED role is shifting to treating more medical and acute cases.

A finding of particular interest in this study is that patients were diagnosed with coronary atherosclerosis at a younger age. The discharge diagnoses of patients older than 65 years were related to cardiovascular diseases in 2009/2010 but those diagnoses were absent in this same age group in 2018/2019; the age density plot showed a shift in coronary atherosclerosis to a younger age (73 to 60 years in 2009/2010 and 2018/2019, respectively). In fact, a study conducted in 2004 showed that the Middle East region holds the highest proportion of diagnosed first myocardial infarction in men aged 40 or younger worldwide.^[31]^ This is especially pertinent in Lebanon, where a recent study on coronary heart diseases in Lebanon showed that heart diseases tend to appear prematurely among Lebanese citizens compared to other developed countries, with 13.4% of Lebanese citizens older than 40 years diagnosed with coronary heart disease.^[32]^ Despite all the efforts by health agencies to mitigate and detect early NCD, the shift of cardiovascular diseases to a younger age is clearly exhibited in our study and it is of high importance to raise awareness, early detection of cardiovascular diseases and mitigation their risk factors CD increased during the last decade, although both years had a bad flu season and the 2018 flu ranked second following the 2009 pandemic of H1N1.^[33]^ Potential causes for the increase in CD could be the waste, electricity, and refugee crises in Lebanon. Repeatedly since 2015, significant swaths of Lebanon suffered from disruptions in waste collection services that led to the accumulation of solid waste in public areas. Studies have shown that inadequate management of waste leads to several health problems and infectious diseases.^[14,34,35]^ Moreover, Lebanon struggles from power outages resulting in poor food hygiene and storage, which is a main risk factor for CD.^[11,13]^ Furthermore, in the past 8 years, Lebanon witnessed a mass population displacement. Total registered refugees increased from 476,045 to 1,392,174 from 2009 to 2019, respectively.^[15]^ This is associated with overcrowding, poor shelter, water, sanitation, and hygiene conditions, increasing the risk factors of CD.^[13]^ Since these 3 risk factors pose significant risk and spread of CD, then public health awareness campaigns, funds, and policies should target both CD and NCDs to mitigate them, especially during complex humanitarian emergencies.

## 5. Conclusion

This study identified the diseases and patients’ characteristics driving the ED visits at the largest tertiary care center in a LMIC. When comparing the 2 study periods, results showed that Lebanon is becoming an aging population and ED visits are shifting from NCD towards CD. Additionally, there is shift of cardiovascular diseases to a younger age. Current national initiatives are targeting NCDs, based on our findings there is also a need to mitigate CD, better allocate resources and improve healthcare workers’ training. Lastly, it is of paramount importance to have focused awareness medical campaigns targeting early detection of cardiovascular diseases and mitigating its risk factors.

This study has several limitations. First, it represents only 1 medical center, it is however the largest medical center in Lebanon with the busiest ED. Secondly, it included 2 timeframes 10 years apart which might have been affected by short term factors, thus its representativeness of the national trends cannot be ascertained. However, the 10-year gap helped set a baseline for future trend measurements in the healthcare system. The data can be used as a potential surrogate to assess the region and national medical status and needs. The data can be used by policymakers as surveillance data to implement preventative and treatment measures especially that national data is not available; Nonetheless future national trend studies are needed to monitor the spread of specific diseases, identifying vulnerable population, evaluate the disease burden, and mitigate them through national policies and awareness campaigns, which can also be applicable in other LMIC.

## Author contributions

**Conceptualization:** Tharwat El Zahran, Najat Saliba, Eveline Hitti.

**Data curation:** Tharwat El Zahran, Lara Ghandour.

**Formal analysis:** Tharwat El Zahran, Lara Ghandour, Anwar Chami, Eveline Hitti.

**Investigation:** Tharwat El Zahran, Lara Ghandour, Anwar Chami, Najat Saliba, Eveline Hitti.

**Methodology:** Tharwat El Zahran, Lara Ghandour, Anwar Chami, Najat Saliba, Eveline Hitti.

**Project administration:** Tharwat El Zahran, Lara Ghandour, Anwar Chami, Najat Saliba, Eveline Hitti.

**Resources:** Tharwat El Zahran, Anwar Chami, Najat Saliba.

**Software:** Tharwat El Zahran, Anwar Chami.

**Supervision:** Tharwat El Zahran, Anwar Chami, Najat Saliba, Eveline Hitti.

**Validation:** Tharwat El Zahran, Lara Ghandour, Anwar Chami, Najat Saliba, Eveline Hitti.

**Visualization:** Tharwat El Zahran, Lara Ghandour, Anwar Chami, Najat Saliba, Eveline Hitti.

**Writing – original draft:** Tharwat El Zahran, Lara Ghandour, Anwar Chami.

**Writing – review & editing:** Najat Saliba, Eveline Hitti.

## Supplementary Material


